# Current Surgical Treatment of Knee Osteoarthritis

**DOI:** 10.1155/2011/454873

**Published:** 2011-04-26

**Authors:** Karolin Rönn, Nikolaus Reischl, Emanuel Gautier, Matthias Jacobi

**Affiliations:** Department of Orthopaedic Surgery, Hôpital Cantonal Fribourg, 1708 Fribourg, Switzerland

## Abstract

Osteoathritis (OA) of the knee is common, and the chances of suffering from OA increase with age. Its treatment should be initially nonoperative—and requires both pharmacological and nonpharmacological treatment modalities. If conservative therapy fails, surgery should be considered. Surgical treatments for knee OA include arthroscopy, cartilage repair, osteotomy, and knee arthroplasty. Determining which of these procedures is most appropriate depends on several factors, including the location, stage of OA, comorbidities on the one side and patients suffering on the other side. Arthroscopic lavage and débridement is often carried out, but does not alter disease progression. If OA is limited to one compartment, unicompartmental knee arthroplasty or unloading osteotomy can be considered. They are recommended in young and active patients in regard to the risks and limited durability of total knee replacement. Total arthroplasty of the knee is a common and safe method in the elderly patients with advanced knee OA. This paper summarizes current surgical treatment strategies for knee OA, with a focus on the latest developments, indications and level of evidence.

## 1. Introduction

Osteoarthritis (OA) of the knee is the commonest joint disorder in the elderly, with a prevalence of about 30% in adults aged >60 years [1]. About half of these subjects will show symptoms such as joint pain, stiffness, effusion and limitation of joint function. With our aging population, the prevalence of OA in the “developed” world is expected to increase. It is anticipated that OA will become the fourth leading cause of disability in the coming decades [2]. 

The etiology of knee OA is multifactorial and includes generalized constitutional factors (e.g., aging, sex, obesity, heredity, and reproductive variables), local adverse mechanical factors (e.g., joint trauma, occupational and recreational abuse, alignment, and postmeniscectomy), and geographic factors. There is a significant genetic component to the prevalence of knee OA, with heritability estimates from twin studies of 0.39–0.65 independent of known environmental or demographic confounders [3]. Genetic variations lead to chondrocyte alterations resulting in osteoarthritis [4, 5].

Diagnostic criteria for OA of the knee include patient history, physical examination, and radiologic and laboratory findings [6]. However, the standard radiograph alone allows in most patients definitive diagnosis of knee OA. Other radiological modalities such as computer tomography, ultrasound imaging, MRI and bone scan can provide alternative or supplementary information [7].

The OA Research Society International (OARSI) has published global, evidence-based, consensus recommendations for the treatment of OA of the hip and knee [8–10]. Of the 51 modalities of treatment addressed in the OARSI recommendations, 35 have been systematically reviewed including a wide range of nonsurgical methods (e.g., physiotherapy, bracing, education, weight reduction, viscosupplementation, corticoid injections, analgesia, other anti-inflammatory treatments, etc.). Initial treatment of knee OA should be conservative. Only if symptoms persist after the appropriate use of nonsurgical treatment, surgery should be considered. Surgical treatment options are arthroscopic debridement, cartilage repair surgery, osteotomy with axis-correction, and unicompartmental or total knee arthroplasty (TKA). We will focus on the latest.

Surgical indication and choice of treatment is based on symptoms (e.g., pain and knee function), OA stage, and patient-related factors such as age, level of physical activity, and patient's comorbidities. Radiological evidence of OA alone (joint space narrowing, osteophytes, etc.) does not justify surgical intervention, which is indicated only in combination with relevant symptoms. Finally, it is the patient's degree of suffering, in correlation to radiological evidence of OA, which determines the time point of surgery. It is important that indication with OA, surgery is always a relative indication. Only in case of progressive knee instability associated to OA surgical treatment (total knee arthroplasty) should not be unnecessary delayed. The choice of surgical treatment, however, underlies in general practice personal, regional, and industry-influenced preferences as indications for different surgical and nonsurgical treatment modalities interfere with each other. 

The present paper will discuss accepted surgical treatment options in knee OA. We focus on the latest developments, indications, and the chosen treatment's efficiency. 

## 2. Surgical OA Treatment

### 2.1. Arthroscopic Lavage and Debridement

Arthroscopic techniques include lavage and debridement of the knee (e.g., shaving of rough cartilage or smoothening of the degenerated meniscus). In theory, arthroscopy for OA should relieve symptoms by removing the debris and inflammatory cytokines that cause synovitis [11, 12]. Debridement can remove torn meniscal fragments and loose cartilage flaps. However the role of arthroscopy in treating knee OA is controversial [8–10]. Although widely used, there is a lack of evidence showing it to have a significant benefit. A controlled trial study by Moseley et al. [13] showed that there was no benefit comparing arthroscopic lavage and debridement with shame surgery. In 2007, Siparsky et al. carried out an evidence-based review of the literature on the arthroscopic treatment of knee OA and found limited support for its use [14]. Dervin et al. [15] showed the importance of patient selection before knee arthroscopy. Patients with evident lesions of the meniscus or cartilage flaps may benefit from surgery. Another study confirms that, in well-selected middle-aged patients with knee arthritis, arthroscopic debridement may be valuable for providing transient relief of symptoms [16]. Patients with less extensive arthritis as seen by radiography, less severe involvement of articular cartilage, and a younger age at the time of surgery have higher probability of improvement [17]. A short duration of pain and mechanical symptoms and mild-to-moderate radiographic stages of arthritis correlate with a better result [14, 18]. However, two recent Cochrane reviews [18, 19] of arthroscopic lavage and debridement for knee OA identified only three well-designed studies [10, 13, 16] and concluded from these that the procedure has no benefit for OA arising from mechanical or inflammatory causes. On the basis of available evidence, arthroscopic lavage seems to provide only short-term benefit to selected patients with mild radiographic OA and effusion. Arthroscopic debridement should not be used as routine treatment for knee OA, although patients with symptomatic meniscal tears and loose bodies with locking symptoms could benefit. 

Quantification of the benefits has been limited by methodological problems and limited analyses in many studies [20]. It is an outpatient procedure with less serious potential complications than other surgical treatments for OA. The postoperative course is predictable, and the risk of complications is acceptably small for most patients. It does not preclude later definitive surgery, and so patient and surgeon may feel it is “worth a try.” Nevertheless, it cannot alter the progression of OA; it may only be a helpful instrument to reduce pain in well-selected patients.

### 2.2. Cartilage Repair Techniques

Damaged articular cartilage has only limited or no healing capacity [21]. Repair of the cartilage surface has therefore been proposed. However cartilage repair is only indicated for focal cartilage defects, which can been seen as a precursor of OA. If the defect is to extended cartilage, repair is no longer indicated. The different techniques can be divided in bone marrow stimulating techniques like abrasion, drilling, or microfracture as well as in replacement techniques like mosaicplasty or osteochondral allograft transplantation and in grafting and combined techniques like periost flap transplantation, and autologous chondrocyte implantation (ACI), autologous matrix induced chondrogenesis (AMIC). 

#### 2.2.1. Bone Marrow Stimulating Techniques

Penetration of the subchondral lamina has been shown to promote cartilage repair tissue; indeed, pluripotent stem cells arising from the subchondral bone marrow may promote chondrogenesis in the defect area. This technique enhances chondral resurfacing and takes advantage of the healing potential of the body. Pridie was the first to describe a technique whereby he used a drill to penetrate the often sclerotic subchondral lamina [22]. In former times, this was provided by an arthrotomy of the joint. Nowadays, it is usually done employing the microfracture technique described by Steadman et al. [23–26]. Using an awl, holes which penetrate 2–4 mm into the subchondral lamina are made at a distance of 3-4 mm from each other. This is relatively simple and can be done arthroscopically. The low-cost and simplicity of this technique have permitted its wide use. The disadvantages of the technique include limited hyaline repair tissue, variable repair cartilage volume, and possible functional deterioration [27].

#### 2.2.2. Osteochondral Transplantation Techniques

Reconstruction of a cartilaginous surface or of osteocartilaginous defects can be done by transplantation of osteochondral grafts. The graft can be autologous or allogenic. Autologous transfer is termed “mosaicplasty” or the osteochondral autologous transfer system (OATS). These terms are used synonymously. It is done by taking one or several cylindrical “plugs” from the peripheries of the femoral condyles at the level of the patellofemoral joint, and the plugs are transferred to the defect with a special cutting devise [28–35]. The procedure can be open (for large defects) or arthroscopic (for small defects) [36]. The advantages of this technique are the use of a bone-cartilage graft consisting of hyaline cartilage, replacing also the often affected underlying bone. Minor integration, limited graft availability and technical difficulties are the disadvantages of the procedure.

#### 2.2.3. Autologous Chondrocyte Implantation (ACI)

**Figure 1:**
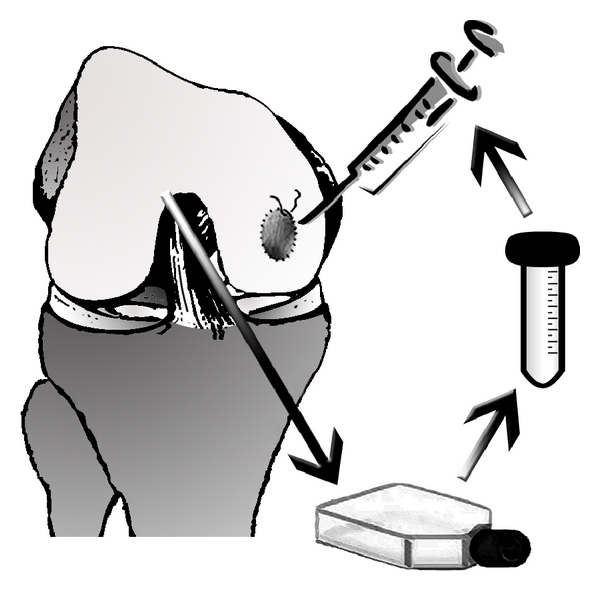
Schematic drawing of autologous cartilage implantation (ACI). The procedure consists of the following steps: (1) cartilage harvest generally performed during arthroscopic surgery, (2) cell culture with expansion of cells in monolayer flasks, and (3) reimplantation of the cells by injecting them underneath a sutured collagen membrane.

In 1994, Brittberg presented the ACI technique whereby cultivated and proliferated autologous chondrocytes are re-implanted underneath a periosteal flap [37]. Chondrocytes are harvested in a first procedure in which a small cartilage probe is taken arthroscopically. The cartilage is then digested and the harvested cells expanded during 3-4 weeks in monolayer culture before implantation (Figure 1). Nowadays, the periost membrane is replaced by a collagen membrane, and cell culture is improved by applying growth factors or by culturing cells in a three-dimensional collagen scaffold which can be directly implanted [38]. The disadvantages of this technique are the two-stage procedure and the costs of the cell culture.

Main indications for cartilage repair techniques are limited size cartilage lesions especially in younger patients. If cartilage damage tends towards an osteoarthritic lesion, cartilage repair procedures are not indicated. Exclusive cartilage repair will not be successful if axial malalignment, ligamentous instability, or patella maltracking is the underlying cause or is associated with the cartilage lesion. Once more one of the key elements of successful surgery is correct indication. Diagnosis is facilitated by improved MRI techniques [39, 40]. Nevertheless, many isolated cartilage lesions are recognized only during arthroscopy [41]. These incidental findings (which are found during arthroscopy or based on MRI) make choosing the correct treatment quite difficult. If bone necrosis is present, debridement and bone grafting must be considered as a concomitant procedure. The use of ACI and other chondral resurfacing techniques is becoming increasingly widespread. The prevalence of symptoms after cartilage repair procedures has been shown to decrease. Randomized controlled trials have been done comparing ACI, microfracture, and mosaicplasty [42–44]. Nevertheless, evidence of a significant difference between ACI and other interventions is lacking [45]. Additional good-quality randomized controlled trials with long-term functional outcomes are required.

### 2.3. Osteotomies around the Knee

Osteotomies around the knee are an accepted method for the treatment of unicompartmental OA with associated varus or valgus deformity. Osteotomies have been carried out since the nineteenth century [46]. Although osteotomies were done regularly in the first half of the twentieth century, the real breakthrough came only with the publications of Jackson, Waugh, Gariépy, Coventry, and others in the late 1950s and 1960s [47–50]. Osteotomy became a standard treatment option for unicompartmental OA of the knee. The classic osteotomy of Coventry was a closed-wedge valgization including a fibula osteotomy and was carried out proximal to the tibial tuberosity [50]. This was the most widely used technique for a long time. In the 1980s and 1990s, osteotomy around the knee lost importance due to the success of knee arthroplasty. Compared with arthroplasty, osteotomy was considered a demanding procedure with an unpredictable outcome and was associated with significant complications. During the last decade, the development of new plates (particularly plates with angular stability) and the tendency for open-wedge osteotomy without bone graft interposition and absence of risk of damage to the peroneal nerve have led to a revival of osteotomy around the knee, particularly for younger patients [51–53].

Osteotomies around the knee alter the weightbearing axis of the lower extremity [54]. The aim is to unload the damaged compartment and to transfer the weight load from the affected areas by slightly overcorrecting into a valgus or varus axis to reduce pain, slow the degenerative process, and delay joint replacement [50, 55, 56].

Fundamental for a satisfactory postoperative outcome is appropriate patient selection, including evaluation of all three knee compartments. The classic inclusion criterion is OA of one compartment in combination with varus or valgus alignment. The femoropatellar compartment should not be affected by OA. Good mobility of the knee is a prerequisite, as well as ligament stability. Instability is not an absolute contraindication because cruciate ligaments can be reconstructed together with correction of the axis [57, 58]. Age is a significant factor to consider. Age >60–65 years is a relative contraindication, whereas biologic age and activity must also be considered. Obesity and chondrocalcinosis are not strict contraindications, but the success rate and prognosis are compromised. Before osteotomy, it is ideal to confirm clinical and radiographic findings by arthroscopy of the knee to ensure that the unaffected compartment is healthy. This can be done in the same procedure.

**Figure 2:**
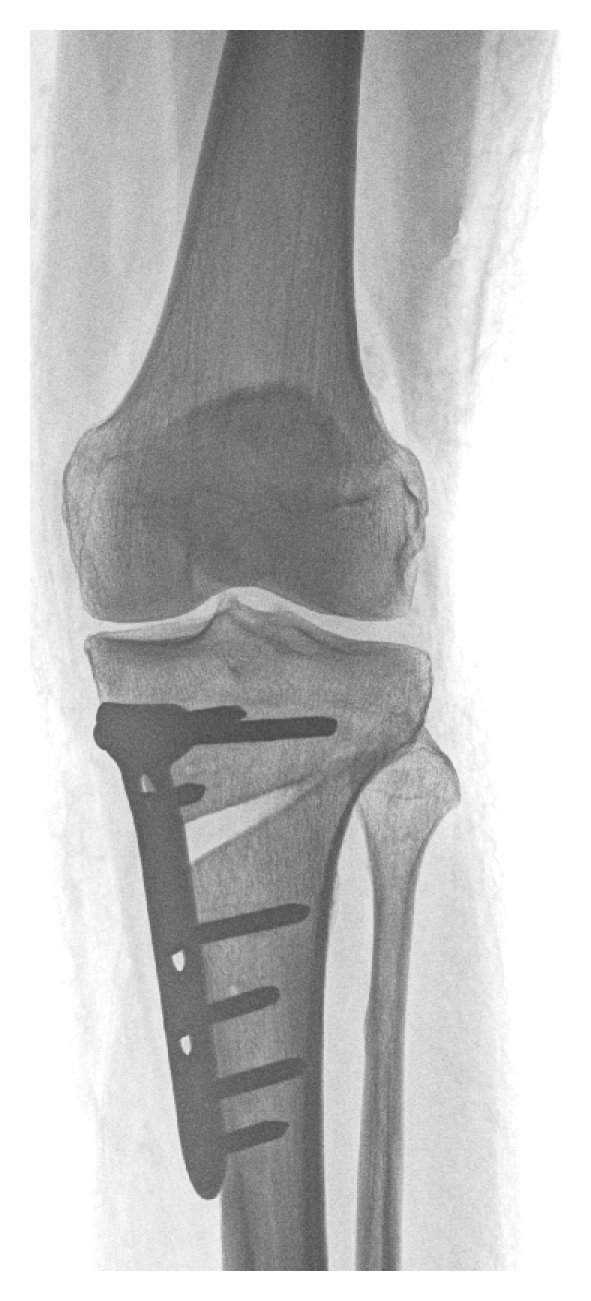
Unloading osteotomy: exemplary a valgisation open-wedge high tibial osteotomy in unicompartmental OA of the medial knee compartment. The corrected position is stabilized by a plate with angular locked screws.

Different techniques are used to correct load axis in unicompartmental knee OA. This includes proximal tibial head osteotomies and supracondylar femoral osteotomies. Both can be done in an additive (open-wedge) or subtractive (closed-wedge) technique, and are regarded as established procedures for the treatment of varus and valgus OA (Figure 2). Valgisation osteotomies are commonly done on the proximal tibia, whereas varisation osteotomies are done on the femoral side. If the deformity is not located near the joint but in the diaphysis of the long bones, the correction should be at the site of the deformity [57].

The classic lateral closing-wedge procedure requires a fibular osteotomy. This is associated with the risk of damage to the peroneal nerve reported to be up to 11% [52]. The joint line has a tendency to end up in an oblique position, which may complicate subsequent placement of the tibial component of a total knee replacement. 

Medial opening-wedge techniques of the tibial head are less demanding, more precise, and faster. This is an advantage, particularly in combined interventions with cruciate ligament reconstruction. Only one saw cut is required, and corrections in the frontal plane can be combined with adjustments in the sagittal plane. New plates with angular stability have been developed during recent years. The increased stability of these plates offers high stability, making bone grafting dispensable [51–53]. The risk to the peroneal nerve is negligible.

Most long-term studies for the closing-wedge valgisation technique have shown good results [59]. Good results are reported during the first years of followup, with deterioration over the time. Insall et al. showed that at the two-year followup, 97% of patients report good results, whereas after five years patient satisfaction decreases to 85%, which dips to 63% after 9 years [60]. Only one Japanese study showed very high survival (90%) after 15 years [61]. Long-term followup for the open-wedge techniques using modern implants with locking screws is not available. Nevertheless, the available mid-term results are promising, and it may become the new standard procedure for valgisation of varus OA.

Osteotomies around the knee are an effective procedure in young and active patients with early OA of one compartment with associated varus or valgus axis. Appropriate patient selection, good preoperative planning, accurate surgical technique, and correct postoperative management can minimize the complication rate and lead to satisfactory outcome. 

Although unloading osteotomies are an accepted and safe treatment modality, no studies have been undertaken to compare it with placebo or conservative treatment alone. However, it has shown to be efficient in reducing pain and improving function [8–10]. Further comparative trials are necessary to define its indication in relation to unicompartmental or total knee arthroplasties.

### 2.4. Joint Arthroplasty

Joint arthroplasty is a well-accepted, safe, and cost-effective method for treatment of advanced knee OA. Owing to its irreversible nature, joint arthroplasty is recommended only in patients for whom other treatment modalities have failed or are contraindicated. Durability of prosthetic components is limited to about 15–20 years but survival of unicompartmental arthroplasties is generally inferior. Therefore arthroplasties should be avoided in patients younger than 60 years whenever possible. If OA is limited to one compartment, unicompartmental knee arthroplasty (UKA) or unloading osteotomy can be considered, otherwise TKA with or without patellar resurfacing is indicated.

#### 2.4.1. Unicompartmental Knee Arthroplasty (UKA)

**Figure 3:**
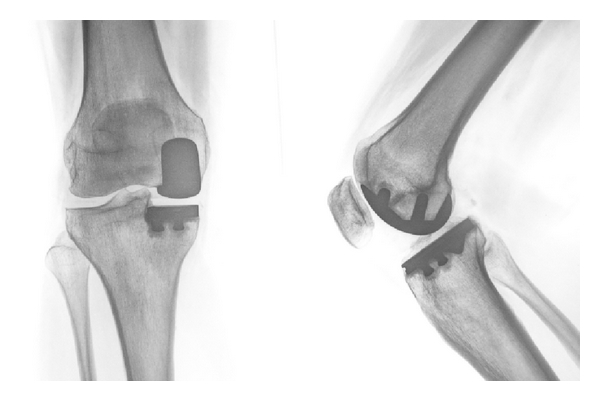
Treatment of an isolated medial compartment OA by unicompartmental arthroplasty.

Since one of the first followup studies reported in the 1970s by Marmor, UKA has received increased interest [62]. UKA is indicated in cases where OA involves only one of the three compartments of the knee: the medial tibiofemoral, lateral tibiofemoral or patellofemoral compartment. The commonest UKA replaces the contact surfaces of the medial tibiofemoral compartment with two metallic prosthetic devices and inserts a polyethylene inlay between them (Figure 3). For successful medial UKA, the initial conditions must provide a well-preserved lateral compartment with respect to meniscus and cartilage [63]. The implant is unrestrained in the sagittal plane, so the stability of the prosthesis depends on intact cruciate ligaments [64]. Considerable malalignment of the limb is a contraindication. Overcorrection to the contralateral compartment must be avoided because it may result in progression of OA and persisting symptoms [65]. Equally, undercorrection is associated with increased likelihood of revision and clinical failure of the UKA [66].

One advantage of UKA includes a less invasive surgical technique [67]. In particular, the patella is not everted and the extensor mechanism is not damaged, permitting a much more rapid recovery and earlier discharge from hospital. It also provides preservation of bone stock, more normal knee kinematics, and greater physiological function [68].

The use of modern implants and surgical techniques has improved the outcome and survival associated with medial UKA [67]. The 10-year survival for medial unicompartmental knee arthroplasty (UKA) is highly variable and ranges from 80.2 to 98% [69, 70]. Anyhow UKA has signifcantly poorer long-term survival than TKA [69]. The target group of UKA differs from that of TKA. UKA is usually done in younger patients with less severe disease, who have better ultimate function, but who wear-out their joints more rapidly. 

Outcomes for the treatment of lateral unicompartmental knee OA are rarely reported [71]. These results are less predictable than those of medial unicompartmental OA, despite recent improvements in implant design. The femoral condyle undergoes a greater translation than the medial condyle during flexion, which may result in instability and dislocation of the tibial insert in mobile-bearing prosthesis [72]. The kinematics of the lateral compartment suggests that a fixed-bearing component may offer a better solution [73].

Isolated femoropatellar OA occurs in 10% of patients with knee OA. Underlying disorders often include prior trauma to the patella, patellar maltracking, trochlear dysplasia, and degeneration secondary to deep bending and overuse. Few patients undergo isolated patellofemoral replacement, although this number is increasing [74, 75]. Failure of isolated femoropatellar arthroplasty is more common than with femorotibial replacements, and the reasons are still not clearly defined. TKA should be considered also for isolated femoropatellar OA, particularly in older patients.

#### 2.4.2. Total Knee Arthroplasty (TKA) (Figure 4)

In advanced knee OA, with more than one compartment involved and failure of conservative treatments, TKA has been shown to be a highly effective treatment that results in substantial improvement in patient functioning and health-related quality of life [76]. Until now it has been the first-line procedure for end-stage knee OA. The long-term results of TKA have been well documented with survival rates of up to 98% at 15 years. [77]. Results in younger patients are mostly reported to be inferior with 76% survival rates at 10 years [78]. 

Although TKA is effective for end-stage arthritis of the knee, postoperative pain occurs or persists in one out of eight patients despite an absence of clinical or radiological abnormalities [79]. The main complications are femoropatellar problems, loosening of components, infections and stiffness of the knee. There is a correlation between existing comorbidities of patients and the range of motion and condition of the knee postoperatively [80]. Nevertheless, there have also been substantial refinements of understanding in the treatment of complications. The importance of patient-related factors to outcome of TKA is shown, and these factors should influence preoperative counseling of patients awaiting TKA. 

One of the central problems in persistent postoperative pain is the femoropatellar joint. However, a general benefit from patella resurfacing has never been proven, and the indications of patella resurfacing are not clearly defined [81, 82]. Complications involving the extensor mechanism and the femoropatellar joint remain the primary noninfectious indications for revision TKA [83]. 

Motivated by the sometimes unsatisfactory results, efforts have been undertaken during recent years to improve the outcome of total knee replacement. These strategies include minimally invasive surgery (MIS), intraoperative control with computer-navigated surgery (CAS) or better instrumentation, improvements in the biomechanic and anatomic design of the implants, and improvements in the fixation of implants.

**Minimal Invasive Surgery (MIS)**

Most knee arthroplasties are implanted through a parapatellar medial arthrotomy with splitting of the quadriceps tendon and the retinaculum/capsule beside the patella and patellar tendon. The patella is usually everted. The so-called “mini-invasive surgery” avoids splitting of the quadriceps tendon. Access is made possible through a mid-vastus approach (splitting of the vastus) or a subvastus approach. Eversion of the patella is avoided. Skin incision is shortened to a minimum. This strategy is thought to have faster recovery times, shorter stays in hospital, fewer problems with patella baja, and improved short-term functional outcomes [83, 84]. Critics have raised questions about malalignment of the leg, malpositioning of the implants, and the length of the learning curve for the procedure [85, 86]. Recent RCTs have failed to show a relevant advantage of this technique [87, 88].

**Biomechanic and Anatomic Improvements in Implant Design**

TKA copies the physiologic biomechanics of the knee joint poorly. The course of motion is defined in the physiologic knee mainly by the cruciate ligaments and in the TKA by the polyethylene inlay. Different types of inlays are available, rotating, fixed-bearing and posterior-stabilized inlays, among others [89]. All of them fail to imitate the original knee motion with rolling back of the femoral condyles on the tibial plateau. Clinical results of different types of inlays are very similar [90]. A newer inlay design, imitating the two cruciate ligaments, is available, but independent long-term followup is lacking [91].

Anatomic implant design has been improved by the following points. First, anatomic studies have revealed that the distal femur is more variable than intended by implant designs. In particular, a difference between male and females can be demonstrated. Implants with a new relationship between the frontal to anteroposterior diameter and adapted Q-angles have been designed. This leads to an expanded implant assortment, but clinical benefit has not been documented by a RCT. A second improvement in implant design is a more anatomically designed trochlea, supporting patella tracking. Third, implants are available which should favor higher flexion of knee prosthesis up to 155° due to higher posterior condyle offset affecting a higher posterior femoral translation and range of flexion [92]. The expected difference between standard knee prosthesis and high flexion prosthesis has not been observed in RCTs [93].

**Implant Fixation**

Cemented fixation of total knee replacement is a standard procedure with good long-term durability. The main advantage of noncemented fixation is the shorter operating time. Whereas clinical outcome shows no significant difference between cemented and noncemented fixation, a recent study found a statistically significant benefit towards improved survival of the cemented compared with noncemented components, with followup ranging from 2 years to 11 years [94]. Another advantage of cemented fixation is that it is less technically challenging because bone cuts do not require a perfect fit with the prosthesis and cement can fill the defects [95]. It is less costly and prevents early migration [96] which may potentially lead to late clinical failures. Cement may also potentially create an effective barrier to polyethylene debris generated from the articular surface, thereby preventing osteolysis and implant loosening [97].

**Intraoperative Control**

A new technology introduced into TKA is computer-assisted surgery (CAS). Computer navigation improves the precision of postoperative alignment after TKA [98]. Despite this effect, patients who underwent navigated TKA did not exhibit improved clinical results at two years when compared with patients who had been managed with conventional TKA. Studies do not reveal early benefit of navigated TKA, and long-term studies are needed to reveal improvements in survival derived from the improvement in limb alignment [99]. Disadvantages are the longer operating time, a learning curve of about 25–30 operations, and the costs of the new technology.

Another new technique relies on patient-specific cutting blocks, which are designed by using the patients MRI or computer tomography as template. These individual cutting blocks allow a precise bone resection adapted to the unique shapes and angles of the joint. Surgery is facilitated, blood loss may be reduced, and the duration of operation is shortened. Disadvantages of this new technology are the additional costs for the cutting block and the fact that the technique relies purely on bony landmarks without paying attention to the ligament balance.

Due to the development of surgical techniques and improved implant technology, the outcome and function of TKA have improved. For successful outcome, good alignment of the tibial and femoral components (as well as correct patella tracking) is essential, leading to lower wear of the prosthesis [100]. TKA has become a successful treatment for advanced and symptomatic knee OA, particularly in elderly patients. Many new developments and designs have been presented and brought to the “medical market” during recent years. They are scientifically interesting and must be followed carefully. Nevertheless, most fall short of proving clinical improvement in the available followup period. Unfortunately, manufacturers regularly misapply these technologies for advertisement purposes, even though evidence of improvement regarding residual pain level, durability of the arthroplasty, and knee function is not present.

## Figures and Tables

**Figure 4:**
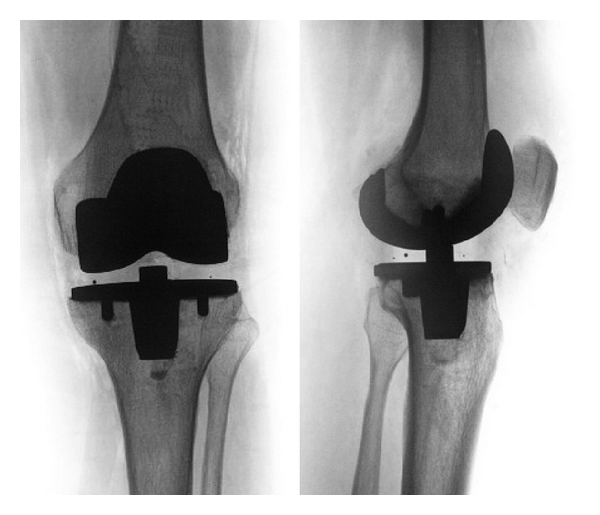
Treatment of advanced knee OA by total knee arthroplasty (example without patella resurfacing).

## References

[1] Felson DT, Naimark A, Anderson J, Kazis L, Castelli W, Meenan RF (1987). The prevalence of knee osteoarthritis in the elderly: the Framingham Osteoarthritis Study. *Arthritis & Rheumatism*.

[2] World Health Organiazation (2002). *The World Health Report 2002: Reducing Risks, Promoting Healthy Life*.

[3] Spector TD, Cicuttini F, Baker J, Loughlin J, Hart D (1996). Genetic influences on osteoarthritis in women: a twin study. *British Medical Journal*.

[4] van der Kraan PM, Blaney Davidson EN, Blom A, van den Berg WB (2009). TGF-beta signaling in chondrocyte terminal differentiation and osteoarthritis. Modulation and integration of signaling pathways through receptor-Smads. *Osteoarthritis and Cartilage*.

[5] Valdes AM, Spector TD, Tamm A (2010). Genetic variation in the SMAD3 gene is associated with hip and knee osteoarthritis. *Arthritis and Rheumatism*.

[6] Altman R, Asch E, Bloch D (1986). Development of criteria for the classification and reporting of osteoarthritis: classification of osteoarthritis of the knee. *Arthritis and Rheumatism*.

[7] Iagnocco A, Meenagh G, Riente L (2010). Ultrasound imaging for the rheumatologist XXIX. Sonographic assessment of the knee in patients with osteoarthritis. *Clinical and Experimental Rheumatology*.

[8] Zhang W, Moskowitz RW, Nuki G (2007). OARSI recommendations for the management of hip and knee osteoarthritis—part I: critical appraisal of existing treatment guidelines and systematic review of current research evidence. *Osteoarthritis and Cartilage*.

[9] Zhang W, Moskowitz RW, Nuki G (2008). OARSI recommendations for the management of hip and knee osteoarthritis—part II: OARSI evidence-based, expert consensus guidelines. *Osteoarthritis and Cartilage*.

[10] Zhang W, Nuki G, Moskowitz RW (2010). OARSI recommendations for the management of hip and knee osteoarthritis—part III: changes in evidence following systematic cumulative update of research published through January 2009. *Osteoarthritis and Cartilage*.

[11] Chang RW, Falconer J, Stulberg SD, Arnold WJ, Manheim LM, Dyer AR (1993). A randomized, controlled trial of arthroscopic surgery versus closed- needle joint lavage for patients with osteoarthritis of the knee. *Arthritis and Rheumatism*.

[12] Ogilvie-Harris DJ, Fitsialos DP (1991). Arthroscopic management of the degenerative knee. *Arthroscopy*.

[13] Moseley JB, O’Malley K, Petersen NJ (2002). Continuing medical education: a controlled trial of arthroscopic surgery for osteoarthritis of the knee. *The New England Journal of Medicine*.

[14] Siparsky P, Ryzewicz M, Peterson B, Bartz R (2007). Arthroscopic treatment of osteoarthritis of the knee: are there any evidence-based indications?. *Clinical Orthopaedics and Related Research*.

[15] Dervin GF, Stiell IG, Rody K, Grabowski J (2003). Effect of arthroscopic débridement for osteoarthritis of the knee on health-related quality of life. *Journal of Bone and Joint Surgery A*.

[16] Hubbard MJS (1996). Articular debridement versus washout for degeneration of the medial femoral condyle: a five-year study. *Journal of Bone and Joint Surgery B*.

[17] Jackson RW, Rouse DW (1982). The results of partial arthroscopic meniscectomy in patients over 40 years of age. *Journal of Bone and Joint Surgery B*.

[18] Rand JA (1991). Role of arthroscopy in osteoarthritis of the knee. *Arthroscopy*.

[19] Laupattarakasem W, Laopaiboon M, Laupattarakasem P, Sumananont C (2008). Arthroscopic debridement for knee osteoarthritis. *Cochrane Database of Systematic Reviews*.

[20] Reichenbach S, Rutjes AW, Nüesch E, Trelle S, Jüni P (2010). Joint lavage for osteoarthritis of the knee. *Cochrane Database of Systematic Reviews*.

[21] Widuchowski W, Lukasik P, Kwiatkowski G (2008). Isolated full thickness chondral injuries. Prevalance and outcome of treatment. A retrospective study of 5233 knee arthroscopies. *Acta Chirurgiae Orthopaedicae et Traumatologiae Cechoslovaca*.

[22] Pridie KH (1959). A method of resurfacing osteoarthritic knee joints. *Journal of Bone and Joint Surgery*.

[23] Steadman JR, Briggs KK, Rodrigo JJ, Kocher MS, Gill TJ, Rodkey WG (2003). Outcomes of microfracture for traumatic chondral defects of the knee: average 11-year follow-up. *Arthroscopy*.

[24] Steadman JR, Rodkey WG, Briggs KK (2002). Microfracture to treat full-thickness chondral defects: surgical technique, rehabilitation, and outcomes. *The Journal of Knee Surgery*.

[25] Steadman JR, Rodkey WG, Briggs KK, Rodrigo JJ (1999). The microfracture technic in the management of complete cartilage defects in the knee joint. *Orthopaedics*.

[26] Steadman JR, Rodkey WG, Rodrigo JJ (2001). Microfracture: surgical technique and rehabilitation to treat chondral defects. *Clinical Orthopaedics and Related Research*.

[27] Mithoefer K, Mcadams T, Williams RJ, Kreuz PC, Mandelbaum BR (2009). Clinical efficacy of the microfracture technique for articular cartilage repair in the knee: an evidence-based systematic analysis. *American Journal of Sports Medicine*.

[28] Hangody L, Feczkó P, Bartha L, Bodó G, Kish G (2001). Mosaicplasty for the treatment of articular defects of the knee and ankle. *Clinical Orthopaedics and Related Research*.

[29] Hangody L, Füles P (2003). Autologous osteochondral mosaicplasty for the treatment of full-thickness defects of weight-bearing joints: ten years of experimental and clinical experience. *Journal of Bone and Joint Surgery A*.

[30] Hangody L, Kárpáti Z (1994). New possibilities in the management of severe circumscribed cartilage damage in the knee. *Magyar Traumatologia, Ortopedia, Kezsebeszet, Plasztikai Sebeszet*.

[31] Hangody L, Kish G, Kárpáti Z, Udvarhelyi I, Szigeti I, Bély M (1998). Mosaicplasty for the treatment of articular cartilage defects: application in clinical practice. *Orthopedics*.

[32] Hangody L, Ráthonyi GK, Duska Z, Vásárhelyi G, Füles P, Módis L (2004). Autologous osteochondral mosaicplasty. *Journal of Bone and Joint Surgery A*.

[33] Hangody L, Vásárhelyi G, Hangody LR (2008). Autologous osteochondral grafting-Technique and long-term results. *Injury*.

[34] Gautier E, Kolker D, Jakob RP (2002). Treatment of cartilage defects of the talus by autologous osteochondral grafts. *Journal of Bone and Joint Surgery B*.

[35] Jakob RP, Mainil-Varlet P, Gautier E (2001). Isolated articular cartilage lesion: repair or regeneration. *Osteoarthritis and Cartilage*.

[36] Hangody L, Kish G, Kárpáti Z, Szerb I, Udvarhelyi I (1997). Arthroscopic autogenous osteochondral mosaicplasty for the treatment of femoral condylar articular defects: a preliminary report. *Knee Surgery, Sports Traumatology, Arthroscopy*.

[37] Brittberg M, Lindahl A, Nilsson A, Ohlsson C, Isaksson O, Peterson L (1994). Treatment of deep cartilage defects in the knee with autologous chondrocyte transplantation. *The New England Journal of Medicine*.

[38] Saris DBF, Vanlauwe J, Victor J (2008). Characterized chondrocyte implantation results in better structural repair when treating symptomatic cartilage defects of the knee in a randomized controlled trial versus microfracture. *American Journal of Sports Medicine*.

[39] Potter HG, Chong LR (2009). Magnetic resonance imaging assessment of chondral lesions and repair. *Journal of Bone and Joint Surgery A*.

[40] Potter HG, Foo LF (2006). Magnetic resonance imaging of articular cartilage: trauma, degeneration, and repair. *American Journal of Sports Medicine*.

[41] Hjelle K, Solheim E, Strand T, Muri R, Brittberg M (2002). Articular cartilage defects in 1,000 knee arthroscopies. *Arthroscopy*.

[42] Gudas R, Stankevičius E, Monastyreckiene E, Pranys D, Kalesinskas RJ (2006). Osteochondral autologous transplantation versus microfracture for the treatment of articular cartilage defects in the knee joint in athletes. *Knee Surgery, Sports Traumatology, Arthroscopy*.

[43] Knutsen G, Drogset JO, Engebretsen L (2007). A randomized trial comparing autologous chondrocyte implantation with microfracture: findings at five years. *Journal of Bone and Joint Surgery A*.

[44] Knutsen G, Isaksen V, Johansen O (2004). Autologous chondrocyte implantation compared with microfracture in the knee: a randomized trial. *Journal of Bone and Joint Surgery A*.

[45] Wasiak J, Clar C, Villanueva E (2006). Autologous cartilage implantation for full thickness articular cartilage defects of the knee. *Cochrane Database of Systematic Reviews*.

[46] Lobenhoffer P, van Heerwaarden J, Staubli A, Jakob RP (2008). *Osteotomie Around the Knee*.

[47] Jackson JP (1958). Osteotomy for osteoarthritis of the knee. Proceedings of the Sheffi eld Regional Orthopaedic Club. *The Journal of Bone and Joint Surgery*.

[48] Jackson JP, Waugh W, Green JP (1961). Tibial osteotomy for osteoarthritis of the knee. *Journal of Bone and Joint Surgery B*.

[49] Gariépy R (1964). Genu varum treated by high tibial osteotomy. In proceedings of the joint meeting of orthopaedic associations. *The Journal of Bone and Joint Surgery*.

[50] Coventry MB (1965). Osteotomy of the upper portion of the tibia for degenerative arthritis of the knee. A preliminary report. *The Journal of Bone and Joint Surgery*.

[51] Lobenhoffer P, Agneskirchner JD (2003). Improvements in surgical technique of valgus high tibial osteotomy. *Knee Surgery, Sports Traumatology, Arthroscopy*.

[52] Staubli AE, De Simoni C, Babst R, Lobenhoffer P (2003). TomoFix: a new LCP-concept for open wedge osteotomy of the medial proximal tibia—early results in 92 cases. *Injury*.

[53] Wagner M, Frenk A, Frigg R (2004). New concepts for bone fracture treatment and the Locking Compression Plate. *Surgical Technology International*.

[54] Maquet PG (1984). *Biomechanics of the Knee: With Applications of the Pathogenesis and the Surgical Treatment of Osteoarthritis*.

[55] Maquet P (1976). Valgus osteotomy for osteoarthritis of the knee. *Clinical Orthopaedics and Related Research*.

[56] Fujisawa Y, Masuhara K, Shiomi S (1979). The effect of high tibial osteotomy on osteoarthritis of the knee. An arthroscopic study of 54 knee joints. *Orthopedic Clinics of North America*.

[57] Aqueskirchner JD, Bernau A, Burkart AC, Imhoff AB (2002). Knee instability and varus malangulation—simultaneous cruciate ligament reconstruction and osteotomy (indication,planning and operative technique, results). *Zeitschrift fur Orthopadie und Ihre Grenzgebiete*.

[58] Paley D, Pfeil J (2000). Principles of deformity correction around the knee. *Orthopade*.

[59] Bonnin M, Chambat P (2004). Current status of valgus angle, tibial head closing wedge osteotomy in medial gonarthrosis. *Orthopade*.

[60] Insall JN, Joseph DM, Msika C (1984). High tibial osteotomy for varus gonarthrosis: a long-term follow-up study. *Journal of Bone and Joint Surgery A*.

[61] Yasuda K, Majima T, Tsuchida T, Kaneda K (1992). A ten- to 15-year follow-up observation of high tibial osteotomy in medial compartment osteoarthrosis. *Clinical Orthopaedics and Related Research*.

[62] Marmor L (1979). Marmor modular knee in unicompartmental disease. Minimum four-year follow-up. *Journal of Bone and Joint Surgery A*.

[63] Murray DW (2000). Unicompartmental knee replacement: now or never?. *Orthopedics*.

[64] Moller JT, Weeth RE, Keller JO, Nielsen S (1985). Unicompartmental arthroplasty of the knee. Cadaver study of the importance of the anterior cruciate ligament. *Acta Orthopaedica Scandinavica*.

[65] Obeid EMH, Adams MA, Newman JH (1994). Mechanical properties of articular cartilage in knees with unicompartmental osteoarthritis. *Journal of Bone and Joint Surgery B*.

[66] Ridgeway SR, McAuley JP, Ammeen DJ, Engh GA (2002). The effect of alignment of the knee on the outcome of unicompartmental knee replacement. *Journal of Bone and Joint Surgery B*.

[67] Borus T, Thornhill T (2008). Unicompartmental knee arthroplasty. *Journal of the American Academy of Orthopaedic Surgeons*.

[68] Boehler NM, Sculco T, Martucci E (2001). Unicompartmental knee arthroplasty. *Knee Arthroplasty*.

[69] Koskinen E, Paavolainen P, Eskelinen A, Pulkkinen P, Remes V (2007). Unicondylar knee replacement for primary osteoarthritis: a prospective follow-up study of 1,819 patients from the Finnish Arthroplasty Register. *Acta Orthopaedica*.

[70] Svärd UCG, Price AJ (2001). Oxford medial unicompartmental knee arthroplasty. A survival analysis of an independent series. *Journal of Bone and Joint Surgery B*.

[71] Sah AP, Scott RD (2007). Lateral unicompartmental knee arthroplasty through a medial approach: study with an average five-year follow-up. *Journal of Bone and Joint Surgery A*.

[72] Gunther TV, Murray DW, Miller R (1996). Lateral unicompartmental arthroplasty with the Oxford meniscal knee. *Knee*.

[73] Ashraf T, Newman JH, Evans RL, Ackroyd CE (2002). Lateral unicompartmental knee replacement. *Journal of Bone and Joint Surgery B*.

[74] Ackroyd CE, Newman JH, Evans R, Edridge JDJ, Joslin CC (2007). The avon patellofemoral arthroplasty: five-year survivorship and functional results. *Journal of Bone and Joint Surgery B*.

[75] Cartier P, Sanouiller JL, Khefacha A (2005). Long-term results with the first patellofemoral prosthesis. *Clinical Orthopaedics and Related Research*.

[76] Buly RL, Sculco TP (1995). Recent advances in total knee replacement surgery. *Current Opinion in Rheumatology*.

[77] Keating EM, Meding JB, Faris PM, Ritter MA (2002). Long-term followup of nonmodular total knee replacements. *Clinical Orthopaedics and Related Research*.

[78] Rand JA, Ilstrup DM (1991). Survivorship analysis of total knee arthroplasty. *Journal of Bone and Joint Surgery A*.

[79] Lundblad H, Kreicbergs A, Jansson KÅ (2008). Prediction of persistent pain after total knee replacement for osteoarthritis. *Journal of Bone and Joint Surgery B*.

[80] Ritter MA, Lutgring JD, Davis KE, Berend ME, Pierson JL, Meneghini RM (2007). The role of flexion contracture on outcomes in primary total knee arthroplasty. *Journal of Arthroplasty*.

[81] Smith AJ, Wood DJ, Li MG (2008). Total knee replacement with and without patellar resurfacing: a prospective, randomised trial using the Profix total knee system. *Journal of Bone and Joint Surgery B*.

[82] Burnett RS, Boone JL, McCarthy KP, Rosenzweig S, Barrack RL (2007). A prospective randomized linical trial of patellar resurfacing and nonresurfacing in bilateral total knee arthroplasty. *Clinical Orthopaedics and Related Research*.

[83] Lonner JH, Lotke PA (1999). Aseptic complications after total knee arthroplasty. *The Journal of the American Academy of Orthopaedic Surgeons*.

[84] Walter F, Haynes MB, Markel DC (2007). A randomized prospective study evaluating the effect of patellar eversion on the early functional outcomes in primary total knee arthroplasty. *Journal of Arthroplasty*.

[85] Berth A, Urbach D, Neumann W, Awiszus F (2007). Strength and voluntary activation of quadriceps femoris muscle in total knee arthroplasty with midvastus and subvastus approaches. *Journal of Arthroplasty*.

[86] King J, Stamper DL, Schaad DC, Leopold SS (2007). Minimally invasive total knee arthroplasty compared with traditional total knee arthroplasty: assessment of the learning curve and the postoperative recuperative period. *Journal of Bone and Joint Surgery A*.

[87] Karachalios T, Giotikas D, Roidis N, Poultsides L, Bargiotas K, Malizos KN (2008). Total knee replacement performed with either a mini-midvastus or a standard approach: a prospective randomised clinical and radiological trial. *Journal of Bone and Joint Surgery B*.

[88] Leopold SS (2009). Minimally invasive total knee arthroplasty for osteoarthritis. *The New England Journal of Medicine*.

[89] Jacobs WCH, Clement DJ, Wymenga AB (2005). Retention versus removal of the posterior cruciate ligament in total knee replacement: a systematic literature review within the Cochrane framework. *Acta Orthopaedica*.

[90] Chaudhary R, Beaupré LA, Johnston DWC (2008). Knee range of motion during the first two years after use of posterior cruciate-stabilizing or posterior cruciate-retaining total knee prostheses: a randomized clinical trial. *Journal of Bone and Joint Surgery A*.

[91] Arbuthnot JE, Brink RB (2009). Assessment of the antero-posterior and rotational stability of the anterior cruciate ligament analogue in a guided motion bi-cruciate stabilized total knee arthroplasty. *Journal of Medical Engineering and Technology*.

[92] Bellemans J, Banks S, Victor J, Vandenneucker H, Moemans A (2002). Fluoroscopic analysis of the kinematics of deep flexion in total knee arthroplasty. Influence of posterior condylar offset. *Journal of Bone and Joint Surgery*.

[93] Kim YH, Choi Y, Kim JS (2009). Range of motion of standard and high-flexion posterior cruciate-retaining total knee prostheses: a prospective randomized study. *Journal of Bone and Joint Surgery A*.

[94] Gandhi R, Tsvetkov D, Davey JR, Mahomed NN (2009). Survival and clinical function of cemented and uncemented prostheses in total knee replacement: a meta-analysis. *Journal of Bone and Joint Surgery B*.

[95] Lombardi AV, Berasi CC, Berend KR (2007). Evolution of tibial fixation in total knee arthroplasty. *Journal of Arthroplasty*.

[96] Nilsson KG, Kärrholm J, Carlsson L, Dalén T (1999). Hydroxyapatite coating versus cemented fixation of the tibial component in total knee arthroplasty: prospective randomized comparison of hydroxyapatite- coated and cemented tibial components with 5-year follow-up using radiostereometry. *Journal of Arthroplasty*.

[97] Freeman MAR, Tennant R (1992). The scientific basis of cement versus cementless fixation. *Clinical Orthopaedics and Related Research*.

[98] Bauwens K, Matthes G, Wich M (2007). Navigated total knee replacement: a meta-analysis. *Journal of Bone and Joint Surgery A*.

[99] Spencer JM, Chauhan SK, Sloan K, Taylor A, Beaver RJ (2007). Computer navigation versus conventional total knee replacement: no difference in functional results at two years. *Journal of Bone and Joint Surgery*.

[100] Collier MB, Engh CA, McAuley JP, Engh GA (2007). Factors associated with the loss of thickness of polyethylene tibial bearings after knee arthroplasty. *Journal of Bone and Joint Surgery A*.

